# Supplementary material for: Dietary calcium intake among Iranian adults: Iranian Multicenter Osteoporosis Study (IMOS-2021)
Source: PLoS One. 2024 Oct 25;19(10):e0310135. doi: 10.1371/journal.pone.0310135 (PMC11508076; doi:10.1371/journal.pone.0310135)
Supplement: S1 Table — (DOCX) [file pone.0310135.s001.docx]

Supplementary material 1. Central tendency measures of dietary calcium intake: Iranian Multicenter Osteoporosis Study (IMOS-2021).

| **Measurement** | **Variable** | | | | | | | | | | | | | | **Total** |
| --- | --- | --- | --- | --- | --- | --- | --- | --- | --- | --- | --- | --- | --- | --- | --- |
|  | **Gender** | | **Age groups** | | **Area of residence** | | **Education** | | | **SES quintiles** | | | | |  |
|  | **Male** | **Female** | **<65** | **≥65** | **Urban** | **Rural** | **No education** | **Diploma or less** | **College or more** | **Poorest** | **Second** | **Middle** | **Fourth** | **Richest** |  |
| Median mg/d (95% CI) | 1067.2 (1007.6-1126.8) | 855.2 (820.5-890.0) | 965.4 (929.1-1001.7) | 869.8 (806.0-933.6) | 957.9 (915.1-1000.8) | 891.4 (820.6-962.2) | 831.1 (775.2-886.9) | 931.2 (889.7-972.7) | 1038.8 (966.4-1111.3) | 817.0 (760.2-873.9) | 889.5 (837.1-941.8) | 982.9 (896.9-1068.8) | 999.5 (936.6-1062.4) | 1029.2 (947.0-1111.3) | 943.5 (910.5-976.4) |
| Mean mg/d (95% CI) | 1180.6 (1126.0-1235.2) | 964.4 (925.5-1003.3) | 1088.2 (1046.2-1130.1) | 1002.3 (951.5-1053.1) | 1072.8 (1034.9-1110.7) | 1033.5 (965.1-1101.8) | 1006.9 (931.3-1082.6) | 1057.3 (1011.0-1103.5) | 1123.6 (1064.4-1182.8) | 921.4 (854.0-988.7) | 978.6 (920.5-1036.8) | 1182.3 (1080.8-1283.9) | 1093.4 (1028.8-1158.0) | 1148.7 (1077.8-1219.7) | 1062.7 (1029.6-1095.8) |
| IQR | 641.1 | 561.4 | 628.7 | 575.3 | 602.1 | 669.8 | 671.7 | 607.8 | 598.9 | 540.7 | 612.9 | 626.1 | 571.6 | 629.6 | 620.2 |
| SD | 590.1 | 477.3 | 566.0 | 476.1 | 530.6 | 574.2 | 593.4 | 540.5 | 489.1 | 466.3 | 475.7 | 599.0 | 478.1 | 512.8 | 542.2 |
| Min. | 68.9 | 46.3 | 46.3 | 162.5 | 190.6 | 46.3 | 46.3 | 162.5 | 230.0 | 46.3 | 185.2 | 230.0 | 301.2 | 304.8 | 46.3 |
| Max. | 3955.9 | 3095.7 | 3955.9 | 3047.5 | 3955.9 | 3314.2 | 3238.7 | 3955.9 | 3231.2 | 2888.5 | 3314.2 | 3955.9 | 3231.2 | 2844.6 | 3955.9 |
| Shapiro-Wilk test of normality ^†^ | **<0.001** | **<0.001** | **<0.001** | **<0.001** | **<0.001** | **<0.001** | **<0.001** | **<0.001** | **<0.001** | **<0.001** | **<0.001** | **<0.001** | **<0.001** | **<0.001** | **<0.001** |

CI: Confidence interval, IQR: Interquartile range, SD: Standard deviation, Min.: Minimum amount of calcium intake (mg/day), Max.: Maximum amount of calcium intake (mg/day), SES quintiles: Socioeconomic status quintiles

† P-value (weighted analysis)
