# Supplementary material for: Dietary calcium intake among Iranian adults: Iranian Multicenter Osteoporosis Study (IMOS-2021)
Source: PLoS One. 2024 Oct 25;19(10):e0310135. doi: 10.1371/journal.pone.0310135 (PMC11508076; doi:10.1371/journal.pone.0310135)
Supplement: S2 Table — (DOCX) [file pone.0310135.s002.docx]

| **Variable** | **Category** | **% Weighted prevalence (95% CI)** | **P*** |
| --- | --- | --- | --- |
| **Gender** | **Male** | 47.8 (43.4-52.3) | **<0.001** |
|  | **Female** | 75.5 (71.9-78.8) |  |
| **Age groups** | **<65** | 60.3 (56.9-63.8) | **0.007** |
|  | **≥65** | 69.0 (63.9-74.0) |  |
| **Area of residence** | **Urban** | 62.1 (58.7-65.5) | 0.325 |
|  | **Rural** | 65.3 (59.9-70.8) |  |
| **Education** | **No education** | 70.2 (64.5-75.4) | **<0.001**^†^ |
|  | **Diploma or less** | 64.5 (60.5-68.4) |  |
|  | **College or more** | 53.3 (47.3-59.1) |  |
| **SES quintiles** | **Poorest** | 75.0 (68.6-80.4) | **<0.001**^†^ |
|  | **Second** | 64.3 (57.6-70.5) |  |
|  | **Middle** | 59.3 (52.4-65.9) |  |
|  | **Fourth** | 58.3 (51.5-64.7) |  |
|  | **Richest** | 56.7 (49.7-63.3) |  |
| **Total** | | 62.9 (60.0-65.7) | |

Supplementary material 2. Weighted prevalence of insufficient dietary calcium intake: Iranian Multicenter Osteoporosis Study (IMOS-2021).

CI: Confidence interval, SES quintiles: Socioeconomic status quintiles

*P-value using proportion test (weighted analysis)

^†^ P for trend using chi-square test (weighted analysis)
